# The relevance of context in memory tasks influences the magnitude of hippocampal remapping

**DOI:** 10.1016/j.celrep.2025.116682

**Published:** 2025-12-11

**Authors:** Gergely Tarcsay, Nicola Masala, Justin D. Yi, Mari K. Igarashi, Usean J. Redic, Laura A. Ewell

**Affiliations:** 1 Anatomy & Neurobiology, School of Medicine, University of California, Irvine, Irvine, CA, USA; 2 Neurobiology of Brain and Behavior, Charlie Dunlop School of Biological Sciences, University of California, Irvine, Irvine, CA, USA; 3 Center for Learning and Memory, University of California, Irvine, Irvine, CA, USA; 4 These authors contributed equally; 5 Senior author; 6 Lead contact

## Abstract

The hippocampus is proposed to associate the context of an episode to its content, facilitating contextual discrimination or generalization between memories depending on the situation. While much is known about how context is encoded by neurons in the hippocampus, little is known about how contextual representations are impacted by disparate cognitive demands. Here, we recorded neuron calcium dynamics during a hippocampal-dependent discrimination/generalization task in which the necessity of attending to context was manipulated. Decoding of context was more robust when context was required to solve the task. Context relevance impacted place cell remapping, with the strongest effects observed in fields near reward locations. Other neuron types, such as non-place cells and directional modulated cells, encoded context to the same extent irrespective of whether context was required. These data suggest that when context is behaviorally important, the hippocampus heightens context specificity, primarily through a place-cell-mediated spatiotemporal code.

## In brief

Tarcsay, Masala, et al. designed a behavioral task that requires mice to either discriminate or generalize between contexts to receive rewards. They report that the extent of hippocampal context representation increases when context is behaviorally relevant (discrimination). Such modulation primarily impacts the spatiotemporal code of place cells.

## Graphical Abstract

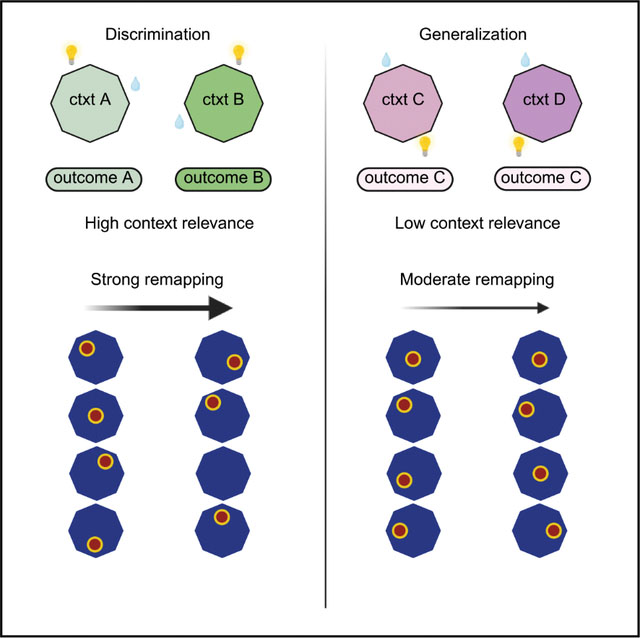

## INTRODUCTION

The hippocampus plays a critical role in forming and retrieving episodic memory.^1–3^ Early theoretical and experimental works proposed the hippocampus as a cognitive map for both spatial location and for the surrounding features of that location,^4–6^ often referred to as context.^7^ The discovery of hippocampal place cells provided a framework in which a given environment is represented as a set of cells with spatially tuned firing patterns,^8,9^ resulting in a population hippocampal spatial code.^10,11^ Hippocampal neurons also respond to non-spatial features of a given environment,^12–14^ supporting the hypothesis of a hippocampal code for contexts.^15–17^ Furthermore, the observation that hippocampal place cell activity reorganizes in distinct environments—known as remapping^18–20^—points to a potential physiological mechanism to create context-specific memories and thus enable context discrimination.^21^ All together, these hippocampal functions allow the association between experience, space, and context of an episode.^22–24^

Although it is often necessary to create memories of distinct contexts, sometimes it is more advantageous to generalize similar experiences to optimize memory load or link experiences together.^25–29^ Several experiments have been designed to investigate hippocampal remapping while rodents were exposed to different contexts, some of which allowed researchers to infer behavioral relevance.^30–38^ However, it remains unclear whether distinct behavioral conditions impact the magnitude of remapping between similar contexts and therefore the extent of context coding. In other words, does the hippocampus represent more detailed contextual information when context-specific memories are required to solve a task compared to when contextual information is behaviorally irrelevant and generalization would be advantageous? To address this question, we developed a novel hippocampus-dependent task in which the same mice observed contextual cues which were either necessary (discrimination) or irrelevant (generalization) to solving the task. Behavioral experiments were combined with freely moving calcium imaging of large populations of CA1 neurons to investigate how hippocampal representations of context may depend on its relevance. Our findings show that the magnitude of context remapping in place cells was increased when the context was behaviorally relevant but that the modulation depended on the prior experience of the mice.

## RESULTS

### Discrimination and generalization memory tasks to test the impact of context relevance on hippocampal remapping

**Figure 1.**
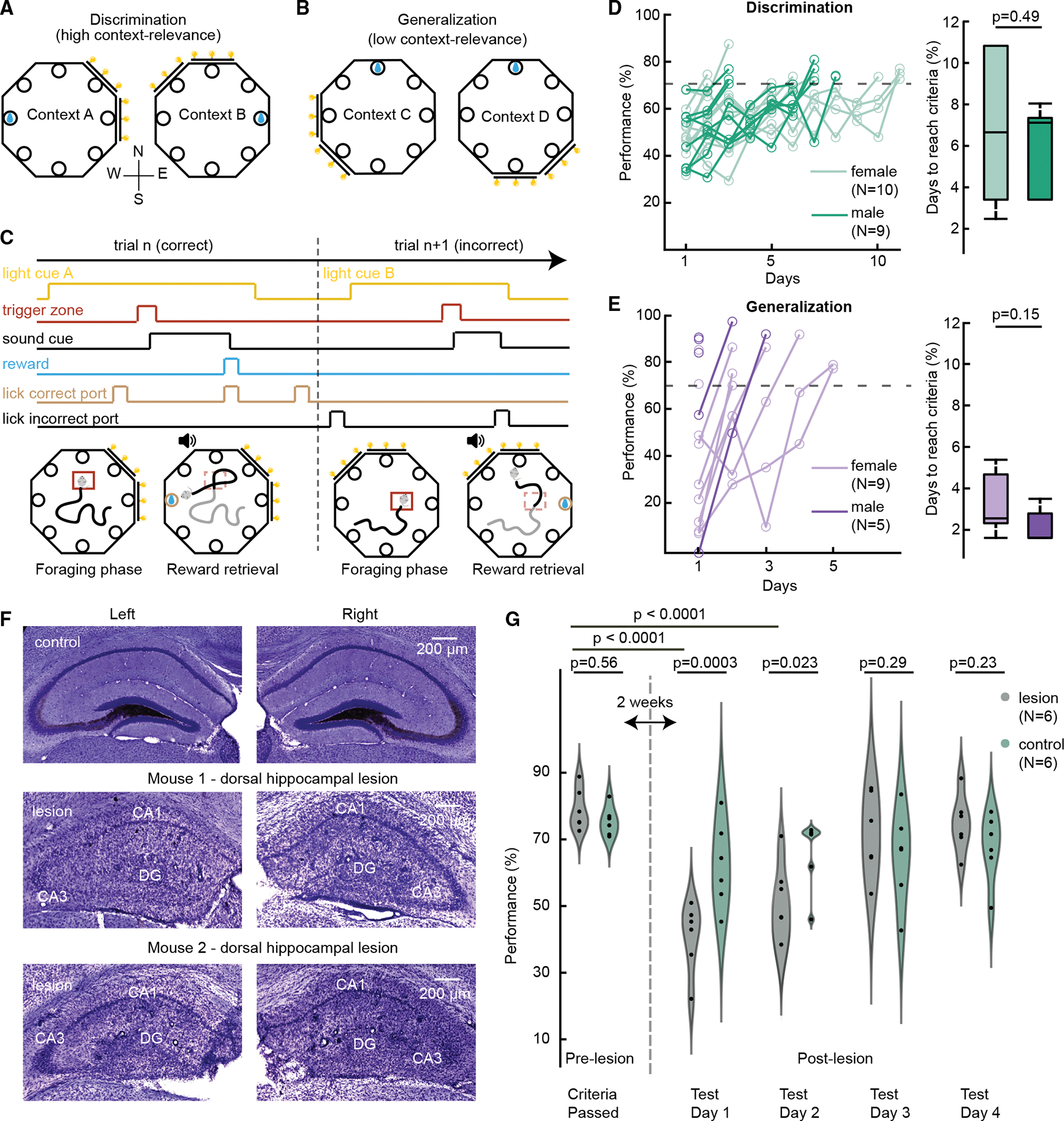
Context-dependent and context-independent memory tasks (A and B) Schematics of the discrimination and generalization paradigms, respectively. Rewarded port is indicated by a droplet; light cue displayed in yellow. Compass rose is for descriptive reference throughout the manuscript and does not indicate true north. During discrimination, mice were trained to discriminate between context A (light cues at E-NE) and context B (light cues at N-NW) to retrieve reward at distinct locations. During generalization, the reward location was fixed across context C (light cues at W-SW) and context D (light cues at S-SE). (C) Schematic of the trial structure displaying a correct (*n*) and subsequent incorrect (*n* + 1) trial. Each trial started with a foraging phase that was terminated once mice ran through a random, hidden trigger zone (red square). Upon trigger zone entry, a sound tone indicated the availability of the reward. If the mouse visited the incorrect reward port, the trial was considered incorrect and terminated. Note that mice were allowed to visit any port during the foraging phase. (D) Learning curves to criteria (70% correct trials, dotted line) did not differ for males and females in the discrimination paradigm (*p* = 0.49, unpaired *t* test). Data are presented in boxplots showing median and interquartile ranges. (E) Learning curves to criteria (70% correct trials, dotted line) did not differ for males and females in the generalization paradigm (*p* = 0.15, unpaired *t* test). (F) Histological examples of dorsal hippocampal lesions. Slices were Nissl stained to visualize cell layers (magenta) and TIMM stained to visualize mossy fibers (brown). One example control mouse (top) and two example lesioned mice (middle, bottom) are shown. (G) Performance of lesion (olive green) and control (light green) group. Mice were trained up to criteria (pre-lesion performance) and then they were injected with either colchicine or saline. Mice were tested 2 weeks after surgery. Performance of the lesion group was significantly lower for the first 2 days and recovered by the third day (repeated measure two-way ANOVA, post hoc Tukey’s multiple comparisons). Individual data points correspond to mice. See also Figure S1 and Tables S1–S4.

To understand how the relevance of context for solving a memory task influences hippocampal representations, we designed a novel context discrimination/generalization task in which we varied the necessity of attending to contextual cues to solve the task (Figures 1A–1C). The task was run in a fully automated, octagonal-shaped arena, where each wall was equipped with a liquid reward port and a strip of LEDs that served as visual cues (STAR Methods, Figures S1A–S1C). Context was defined as the mixture of global and local cues in which the light cues served as variable components (see Rudy et al.^7^). Pseudo-randomly varying the location of light cues allowed us to toggle between contexts. In the context-dependent discrimination paradigm, mice were trained to discriminate between two contexts to retrieve reward at distinct locations (Figure 1A). In context A, light cues (e.g., at the northeast-east walls of the arena) indicated reward availability at a specific location (e.g., at the west wall), while in context B, light cues adjacent to those in context A (e.g., at the northwest-north walls) indicated a different reward location (e.g., at the east wall). Thus, to correctly retrieve rewards, mice had to learn context to reward-location associations and importantly to discriminate between similar contexts (context A and context B). Mice (10 female; 9 male) learned to discriminate (≥70% correct trials) in 2–11 days, and we did not observe a sex difference (Figure 1D, females: 6.8 ± 3.8; males: 5.8 ± 2.2; mean ± SD days; *p* = 0.49; t_17_ = 0.7; unpaired *t* test). Some mice performed close to criteria already on the first day due to a pre-training protocol comprising guided trials that preceded the training phase (see STAR Methods for details, Figure S1D). In the context-independent generalization paradigm, mice learned to generalize across similar contexts (context C and context D) and associate them both with one common reward location (Figure 1B), thus removing the need to discriminate between contexts to receive rewards. Mice (9 female, 5 male) learned the generalization paradigm (≥70% correct trials) in 1–5 days, and again we did not observe a sex difference (Figure 1E, females: 2.8 ± 1.6; males: 1.6 ± 0.9; mean ± SD days; *p* = 0.15; t_12_ = 1.5; unpaired *t* test). To eventually investigate hippocampal spatial representation of contexts during experiments, dense spatial coverage of the arena is essential. Thus, the tasks described earlier employed a trial structure similar to that presented in Morales et al.^39^ Each trial started with a foraging phase in a given context, when mice were searching for a random hidden trigger zone (Figure 1C). When the zone was found, a sound tone indicated reward availability. If the mouse visited the correct port (indicated by the context), a sucrose drop was delivered as a reward. If they instead visited another port, the trial was considered incorrect and no reward was given. Finally, if the mouse failed to visit any port within 1 min from the onset of the sound tone, the trial was ended and considered incorrect. Port visits during the foraging phase were allowed. Within each paradigm (discrimination or generalization), context A/B or C/D trials were pseudo-randomized.

### The role of the hippocampus in contextual discrimination

To test hippocampal involvement in the discrimination paradigm implemented in our task, dorsal hippocampi of mice that had been trained to criteria were injected with colchicine (*n* = 6) to induce cell death or injected with saline (*n* = 6) as a sham control (Figures 1F and S1E). Although colchicine has been shown to preferentially lesion the dentate gyrus^40^ (DG), we observed extensive cell loss in CA3 and CA1 sub-regions as well, and thus we considered the colchicine manipulation as a full dorsal hippocampal lesion (Figure S1F, one-way ANOVA; *p* = 0.0005, F_2_ = 13.37; DG: 2.9% ± 1.9%; CA3: 15.8% ± 6.2%; CA1: 12.4% ± 4.4%; mean ± SD relative volume; p_DG-CA3_ = 0.0004; p_DG-CA1_ = 0.006; p_CA3-CA1_ = 0.4; post hoc Tukey’s multiple comparisons; Tables S1 and S2). Two weeks after lesioning, mice were tested on discrimination for 4 days (Figure 1G). We found a significant difference between the lesion and control groups (repeated measure two-way ANOVA; *p* = 0.0001; F_4,40_ = 7.67; for the interaction term; Table S3) due to impaired performance of the lesion group on the first 2 days (lesion: day 1, 41% ± 11% and day 2, 53% ± 11%; control: day 1, 63% ± 13% and day 2, 67% ± 11%; mean ± SD performance; *p* = 0.0003 and *p* = 0.023; post hoc Tukey’s multiple comparisons test, Table S4). Performance of the lesion group recovered by the third day (lesion: day 3, 72% ± 13% and day 4, 75% ± 9%; control: day 3, 66% ± 14% and day 4, 68% ± 10%; *p* = 0.29 and *p* = 0.23). To further understand the behavioral recovery, we classified incorrect trials into three categories: (1) context error (when the visited port was rewarded in the other context), (2) spatial precision error (when the visited port was adjacent to the reward port), and (3) non-specific error (when the visited port was any other port not defined as the other two error types) (Figure S1G). We calculated a baseline composition of errors from mice with no manipulation (*n* = 22 mice) and found that trained mice primarily made context errors. We found that the lesion group significantly deviated from baseline even when their performance recovered, making more spatial precision and non-specific errors than control mice (*p* < 0.0001 on day 1; *p* = 0.0037 on day 2; *p* = 0.0002 on day 3; *p* = 0.012 on day 4; lesion: χ^2^ = 48.6; 14; 19.4; 11.7; control: χ^2^ = 4.6; 1; 8.5; 3.3; day 1; day 2; day 3; day 4; df = 4; chi-squared test with Bonferroni correction). The intact ventral hippocampus may be critical for performance recovery as neurons there are also spatially modulated, but with larger firing fields.^41,42^ Alternatively, mice could have developed a hippocampal-independent strategy.^43–45^ Both cases would be consistent with increased spatial precision errors, as the most precise codes for space reside in the dorsal hippocampus. Next, we utilized this task to test how the hippocampal representation of contexts depends on discrimination or generalization of contexts.

### Population codes depend on paradigm type

A recent theory in the learning and memory field is that contexts are encoded in distinct temporal patterns of co-active neuronal ensembles in the hippocampus.^33,46^ Using a supervised machine learning approach, we asked whether contexts can be decoded from CA1 neuronal calcium activity and whether decoding accuracy of contexts depends on the relevance of context for solving the task. In other words, are contexts represented more distinctly in neuronal ensembles when the context is behaviorally relevant (i.e., in the discrimination paradigm), compared to when context is not behaviorally relevant (i.e., in the generalization paradigm)?

**Figure 2.**
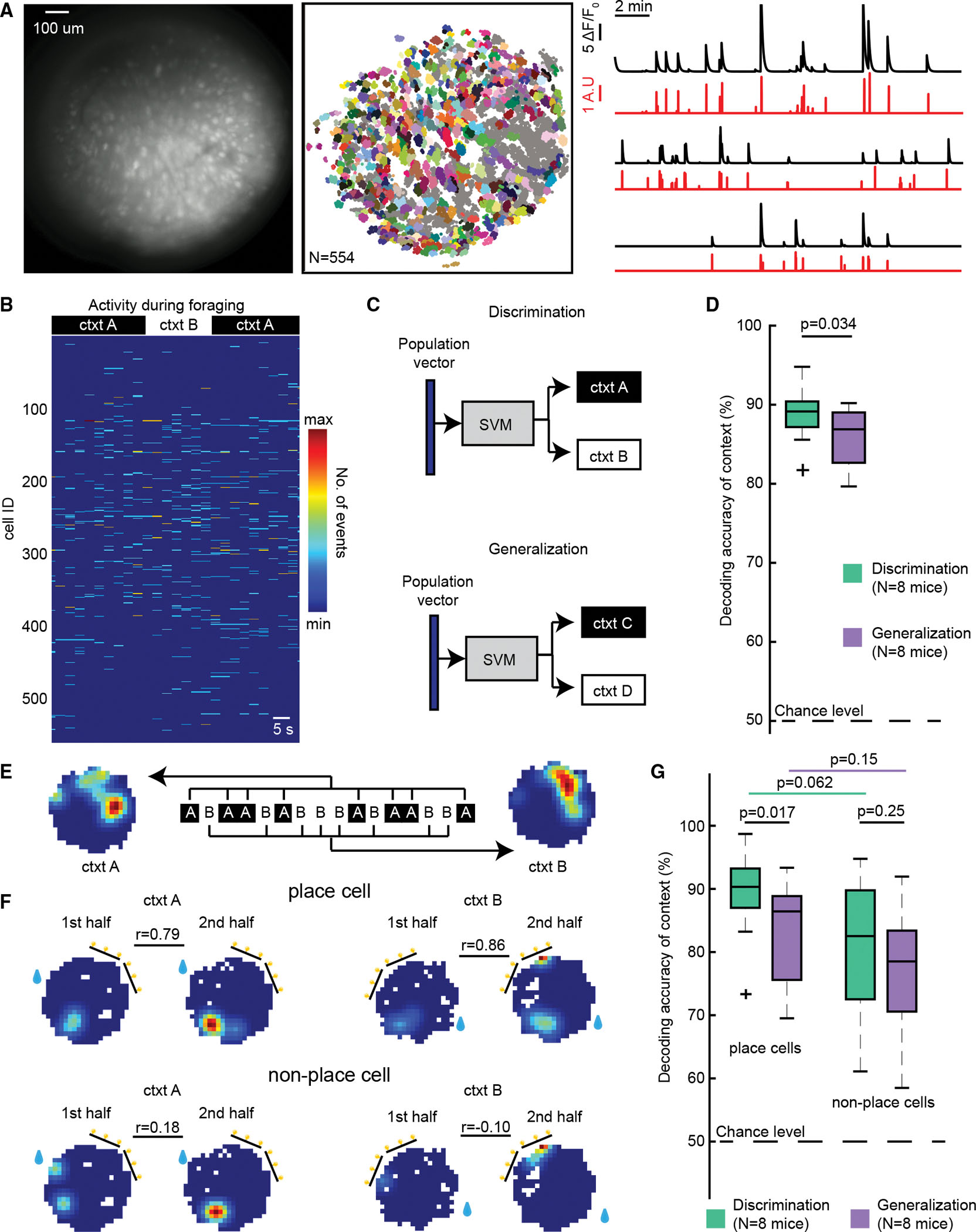
Population code for contexts is more robust when context is necessary for solving the task (A) Example field-of-view of hippocampal CA1 neurons (left), extracted spatial footprints (middle), and their corresponding calcium traces (right, black) and deconvolved signals (right, red). Only neurons with stable calcium dynamics (colored footprints) were included for analysis. (B) Example of smoothed and binned (3 s) calcium activity of CA1 neurons from one mouse across the foraging periods from three consecutive trials in the discrimination paradigm. (C) Temporal population vectors were used to train a support vector machine (SVM) to decode context. The decoder was trained on the discrimination (top) and on the generalization (bottom) paradigms for each mouse. (D) Decoding accuracy of context was compared between discrimination (green) and generalization (purple) (*p* = 0.034, paired *t* test). Data are presented in boxplots showing median and interquartile ranges. Outliers are marked by crosses. (E) For each neuron, context maps were constructed. Context A trials were concatenated to construct a “context A” map, and context B trials were concatenated to construct a “context B” map. The same procedure was done for generalization for contexts C and D. (F) Maps were then split into the first and second half of the session. Spatial correlation coefficients (r) between half maps were calculated. Cells were considered as place cells if the measured stability (r) across the first and second half of the session was significant from a shuffled null distribution in at least one context. (G) Decoding accuracy for context by place cell and non-place cell subpopulations in the discrimination and generalization paradigms (repeated measures model, post hoc Tukey’s multiple comparisons). See also Figures S2 and S3 and Tables S5–S8.

To balance for training history across the two paradigms, one cohort of mice (G1, *n* = 1 female, *n* = 3 male) was trained in the generalization paradigm first, and a second cohort of mice (D1, *n* = 3 female, *n* = 2 male) was trained in the discrimination paradigm first (Figure S1D). Throughout, calcium events from individual neurons were imaged in dorsal CA1 (Figures 2A and S2A–S2C). Next, a support vector machine (SVM) was trained for each mouse to decode context from binned population activity of the full population of neurons recorded (Figures 2B and 2C). The analysis was restricted to the foraging period of the trials (to exclude reward consumption-related activity) and included both locomotive and resting periods. We found that contexts were decoded with high accuracy in both paradigms, revealing that ensembles carry context information even when the task did not require the mice to discriminate between contexts (Figure 2D; discrimination: *n* = 8; 88.27% ± 3.26%; generalization: *n* = 8; 86.29% ± 3.93%; mean ± SEM accuracy; Tables S5 and S6). We also found a moderate but significant reduction in the decoding accuracy in the generalization paradigm compared to the discrimination paradigm (mean performance difference = 1.98% ± 0.76%; *p* = 0.034, t_7_ = 2.6, paired *t* test). This result was not driven by a general difference in locomotive behavior in the two paradigms. We found no mean difference in median running speeds (1.4 ± 0.7 cm/s, mean ± SEM; *p* = 0.09, t_7_ = 1.97, paired *t* test, *n* = 8). There was also no difference in the proportion of resting time (2.3% ± 3.2%; *p* = 0.5, t_7_ = 0.7, paired *t* test) between the two tasks.

We next sought to understand how place cells, which are known to be important for context coding,^17^ contributed to the observed decoding result. We identified place cells as neurons with significantly stable spatial tuning at least in one context (STAR Methods, Figures 2E and 2F) and restricted the decoding analysis to either place cell or non-place cell populations. We found a significant context relevance modulation of place cells (Figure 2G; mean performance difference = 3.63% ± 1.17%; *p* = 0.017, repeated measures model and post hoc Tukey’s multiple comparisons; Tables S7 and S8) but not for non-place cells (Figure 2G; mean performance difference = 1.34% ± 1.06%; *p* = 0.25). In the discrimination paradigm, decoding trended toward being stronger in place cells compared to non-place cells (mean performance difference = 6.88% ± 3.10%, *p* = 0.062), and in generalization, decoding accuracy was similar in both cell types (mean performance difference = 4.59% ± 2.84%, *p* = 0.25). Taken together, decoding accuracy is sensitive to paradigm type for the place cell population only.

Mice performed significantly better on the generalization paradigm than on the discrimination paradigm (Figure S3A; *p* < 0.0001, t_7_ = −10.8, paired *t* test), so we asked whether behavioral performance difference explains the distinct decoding accuracy in the two paradigms. We trained the SVM exclusively on correct trials such that, in each paradigm, 100% behavioral performance was simulated, and we constrained decoding to using the place cell population only. We found that the decoding accuracy was similar to the case when incorrect trials were included as well (Figure S3B; discrimination: performance difference = 1.03% ± 0.56%; *p* = 0.22, t_7_ = 1.8; generalization: performance difference = 0.15% ± 0.27%; *p* = 1, t_7_ = 0.5; paired *t* test with post hoc Bonferroni correction). We further found context decoding accuracy was significantly higher in the discrimination paradigm compared to generalization (Figure S3C; performance difference = 2.86% ± 0.78%; *p* = 0.008, t_7_ = 3.6, paired *t* test). Altogether, these data indicate that although context is always encoded, when context was necessary for solving the task, the representations are more robust, are more strongly represented in place cells, and do not depend on differences in behavioral performance.

### Spatial remapping of place cells between contexts depends on paradigm type and past experience

**Figure 3.**
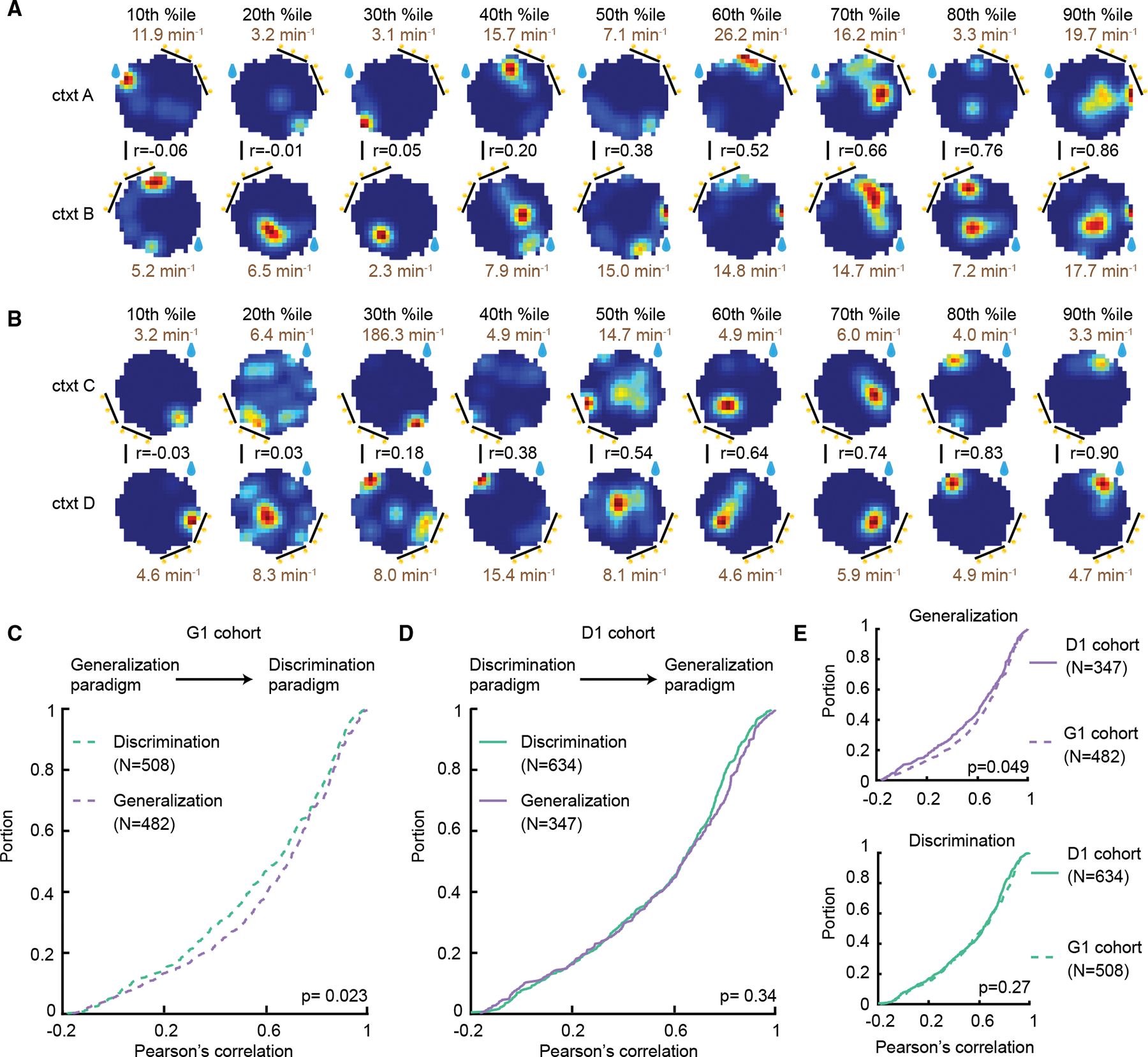
Context relevance impacts the magnitude of spatial remapping of place cells in an experience-dependent manner (A) Place maps constructed from contexts A and B in the discrimination paradigm are shown for nine place cells. Remapping between contexts was quantified by calculating the Pearson’s correlation coefficient (r), and displayed cells were sorted from low to high correlation coefficient percentile (%ile). Peak event rate is displayed in brown text. (B) Same as in (A) but for generalization. (C and D) Cumulative distribution of spatial correlation between contexts for the G1 (learned generalization first and then discrimination) and D1 (learned discrimination first and then generalization) cohorts, respectively. Note that only the G1 cohort exhibits a context relevance modulation of the magnitude of remapping (*p* = 0.023, 0.34, Wilcoxon rank-sum test). (E) Comparison between cohorts for remapping in the generalization (top) and in the discrimination (bottom) paradigms (*p* = 0.049, 0.27, Wilcoxon rank-sum test).

The observation that the decoding accuracy of context from place cells differed between discrimination and generalization paradigms motivated us to further dissect the mechanisms in the spatial domain. A common theory is that remapping of place cells between contexts^4,5,17^ is the neural mechanism of context coding. To test whether the magnitude of remapping was sensitive to paradigm type, for each neuron, we constructed place maps for the two contexts (A/B for discrimination and C/D for generalization) and spatial correlations between the maps were calculated (Figures 3A and 3B). Spatial remapping has been shown to be impacted by past experience,^18,47–50^ so we separated neurons by cohort (G1 and D1). Interestingly, we found that the G1 cohort exhibited a difference in remapping depending on context relevance (Figure 3C, discrimination: 0.64 [0.36–0.82], median [25th percentile–75^th^ percentile]; generalization: 0.69 [0.44–0.85]; *p* = 0.023, z = −2.28, Wilcoxon rank-sum test). In contrast, the extent of remapping was similar in the two paradigms for the D1 cohort (Figure 3D, discrimination: 0.64 [0.34–0.79]; generalization: 0.64 [0.35–0.82]; *p* = 0.34, z = −0.96, Wilcoxon rank-sum test). To further understand the experience effect, we focused on each paradigm separately and compared cohorts. For generalization, remapping was significantly reduced in the G1 cohort, i.e., in the mice that learned the generalization paradigm first (Figure 3E, top, *p* = 0.049, z = −1.97, Wilcoxon rank-sum test). For discrimination, we observed no difference in the magnitude of remapping between the two cohorts (Figure 3E, bottom, *p* = 0.27, z = −1.1, Wilcoxon rank-sum test). Together, these results indicate that when mice first learn to generalize between contexts, the hippocampal maps for each context are more similar to each other. Then, upon learning the rule in which context discrimination is necessary, the extent of remapping increases. On the other hand, when context discrimination is learned first, the high level of remapping is inherited to the generalization paradigm even though context could be ignored in that case. Finally, these results underscore that strong remapping during the discrimination paradigm is robust regardless of prior experience.

### Place field rate coding for contexts is impacted by paradigm type in an experience-dependent manner

**Figure 4.**
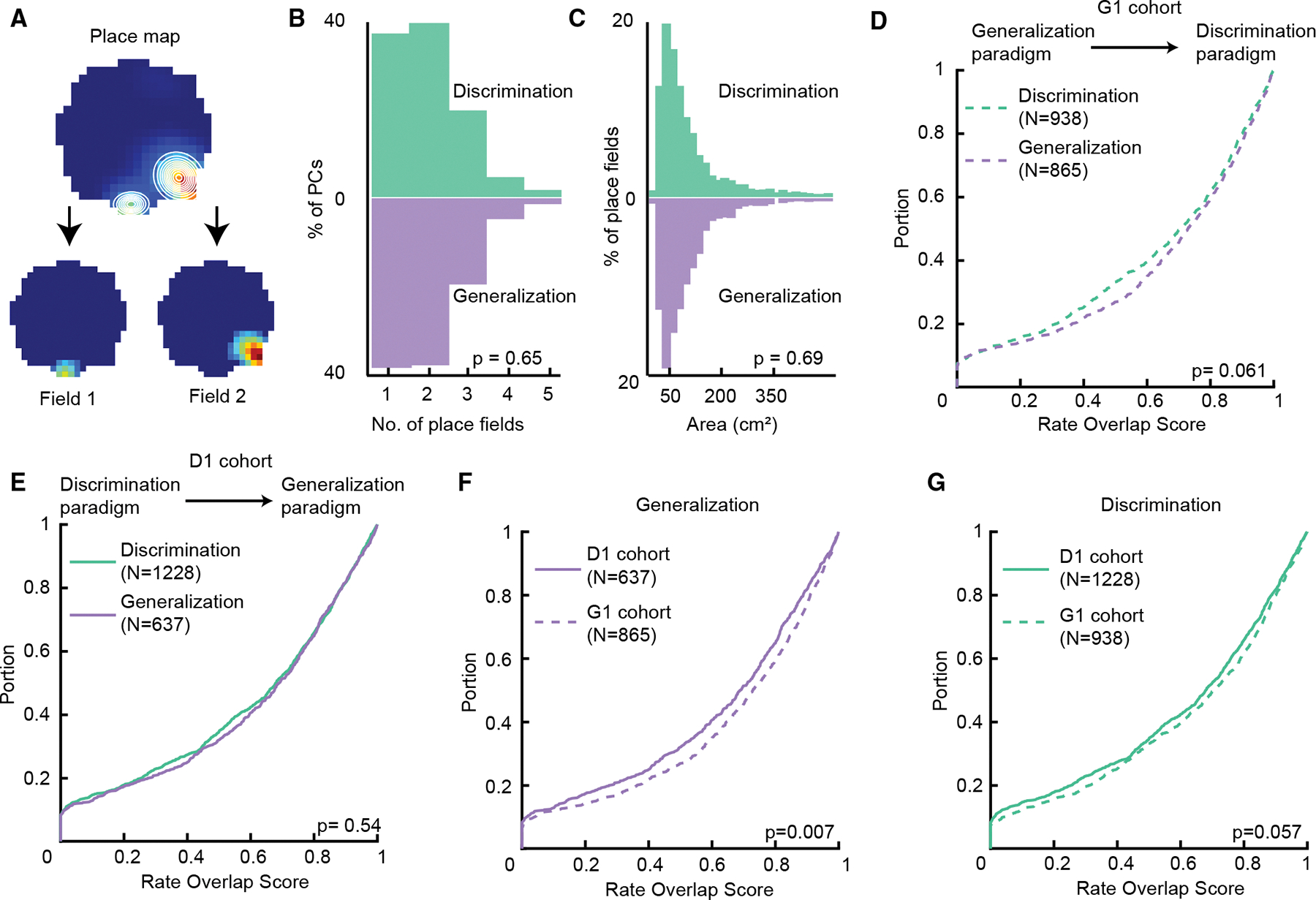
Impact of context relevance and experience on place field rate remapping (A) To extract place fields, place maps were fit with a multi-component Gaussian mixture model (white). The number of components was determined by the number of rate peaks (in this example, two). (B and C) Histogram of number of extracted place fields and place field size, respectively, for discrimination (top) and generalization (bottom) (*p* = 0.65, 0.69, Wilcoxon rank-sum test). (D and E) Cumulative distribution for the rate overlap score between context in discrimination and generalization for the G1 and D1 cohorts, respectively (*p* = 0.061, 0.54, Wilcoxon rank-sum test). (F) Comparison between cohorts of the rate overlap distributions in the generalization paradigm (*p* = 0.007, Wilcoxon rank-sum test). (G) Same as in (F) but for the discrimination paradigm (*p* = 0.057, Wilcoxon rank-sum test).

We next asked whether paradigm-dependent differences in context coding are additionally expressed in rate remapping. Rate remapping, which has previously been implicated in context coding,^13,38^ is another type of partial remapping in which the spatial location of place fields is preserved and only the activity rate changes. Although more commonly studied in single-unit studies, rate remapping has also been observed in calcium imaging experiments.^51^ To account for the contribution of individual place fields to rate coding, we separated place fields by fitting a k-component Gaussian mixture model on each place map, where k is the number of place fields of a given place cell (STAR Methods, Figure 4A). Field numbers and field sizes of place cells did not differ between the two paradigms (Figures 4B and 4C; number of place fields, discrimination: 2 [1–2]; median [25th percentile–75th percentile]; generalization: 2 [1–2]; field size, discrimination: 40 [25–65] cm^2^; generalization: 41 [26–65] cm^2^; *p* = 0.65, z = 0.45; *p* = 0.69, z = −0.4; Wilcoxon rank-sum test). To quantify rate remapping, we calculated the rate overlap score between the two contexts for each place field. Low rate overlap corresponds to stronger rate remapping. Similar to the spatial correlation results, for the G1 cohort, we observed a trend of stronger rate remapping in the discrimination paradigm (Figure 4D, discrimination: 0.70 [0.40–0.88]; generalization: 0.73 [0.46–0.89]; *p* = 0.061, z = −1.88; Wilcoxon rank-sum test). The difference in rate overlap was significant when the statistical test was not restricted to the median (*p* = 0.015; k = 0.07; Kolmogorov-Smirnov test). We found similar rate overlaps in the two paradigms for the D1 cohort (Figure 4E, discrimination: 0.68 [0.35–0.86]; generalization: 0.69 [0.40–0.86]; *p* = 0.54, z = −0.61; Wilcoxon rank-sum test). These results suggest a moderate context relevance modulation of rate coding that also depends on past experience, similar to spatial coding. Next, we compared rate overlap between contexts in generalization and discrimination, respectively, for the two cohorts to gain insight into the experience-dependent effect (Figures 4F and 4G). For generalization, the G1 cohort showed less rate remapping between contexts (*p* = 0.007, z = −2.71; Wilcoxon rank-sum test). For discrimination, there was a trend of more rate remapping in the D1 cohort (*p* = 0.057, z = −1.9, Wilcoxon rank-sum test), indicating that past experience primarily affects rate remapping in generalization in the G1 cohort. Again, similar to the spatial correlation result, when animals first learn to generalize, fields remap less between the two contexts, and then the extent of remapping increases in the discrimination paradigm.

### Magnitude of rate remapping of reward site place fields between contexts is modulated by paradigm type but not experience

**Figure 5.**
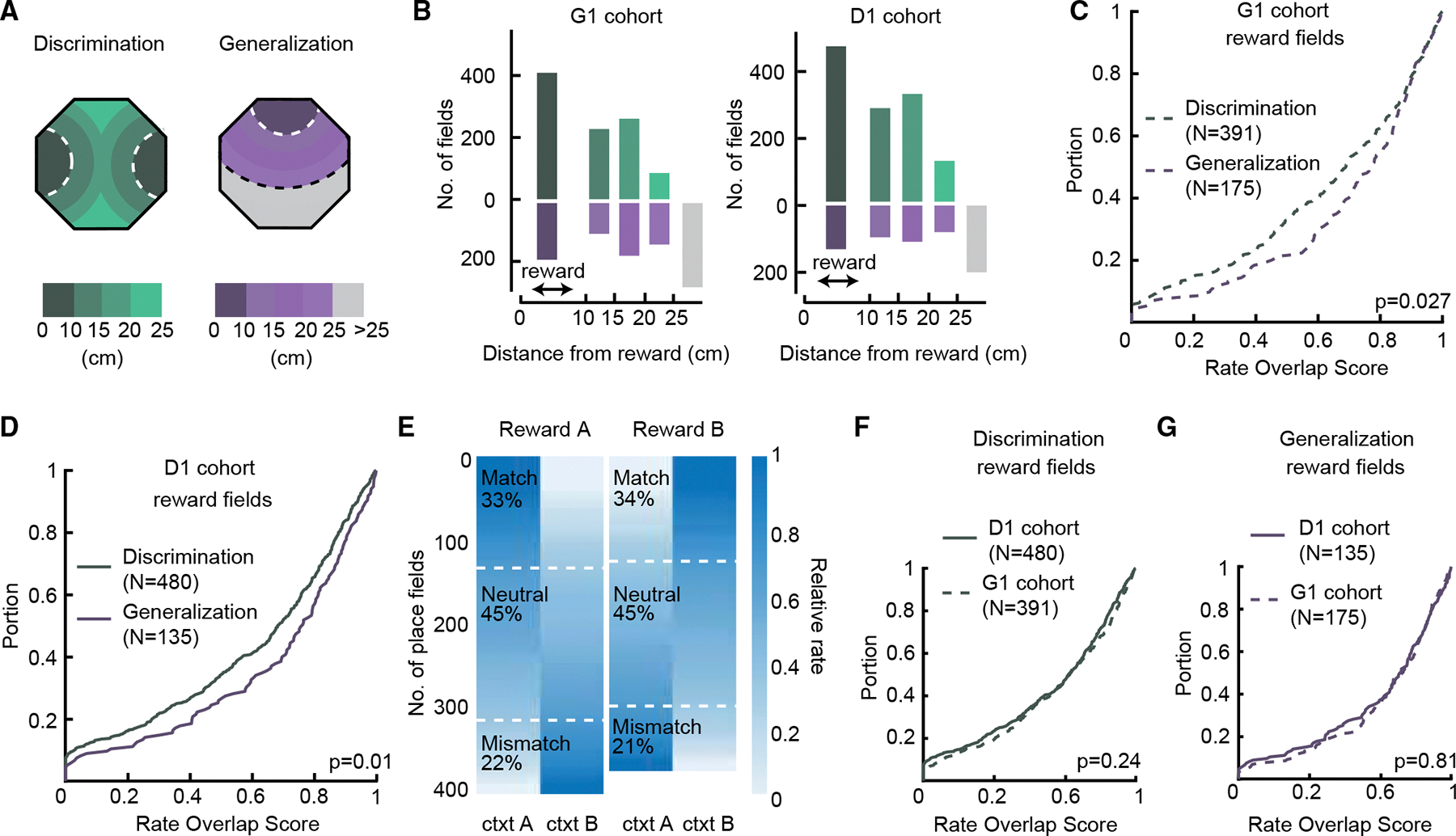
Rate remapping of reward fields is modulated by context relevance but not experience (A) Place fields were sorted based on their distance from reward locations in discrimination (left) and generalization (right). Within a 10 cm radius (white dashed line), fields were considered reward fields. Otherwise, fields were divided into 5 cm bins (beyond white dashed line). Note that in the generalization paradigm, fields further than 25 cm were present (gray) but not in discrimination due to two reward locations. (B) Number of place fields in each bin for the D1 (left) and G1 (right) cohorts in discrimination (green) and in generalization (purple). (C and D) Cumulative distributions of reward field rate overlap scores between contexts for the discrimination and the generalization paradigms for the G1 and D1 cohorts, respectively. Note that context relevance impacts the magnitude of rate remapping in both cohorts (*p* = 0.027, 0.01, Wilcoxon rank-sum test). (E) Activity of reward fields was classified as (1) match fields, e.g., increased activity at reward 1 in context A; (2) neutral fields, i.e., reward activity is independent from context; and (3) mismatch fields; e.g., increased activity at reward 1 activity in context B. (F and G) Comparison of reward field rate remapping between D1 and G1 cohorts in the generalization (F) and discrimination (G) paradigms (*p* = 0.24, 0.81, Wilcoxon rank-sum test).

Given that both paradigms are context-reward association tasks, we asked whether place field activity at reward locations exhibits context-relevant modulation. We classified reward fields as those with field centers within 10 cm of rewarded ports (STAR Methods, Figures 5A and 5B). There was a higher number of reward fields in the discrimination paradigm compared to the generalization paradigm, which was consistent with the discrimination paradigm having twice the number of rewarded locations (Figure 5B). For both cohorts, there was significantly more rate remapping between contexts in the discrimination paradigm compared to the generalization paradigm, suggesting that context relevance strongly impacts reward fields (Figures 5C and 5D; G1 cohort; discrimination: 0.69 [0.43–0.88], median [25th percentile–75th percentile]; generalization: 0.77 [0.58–0.90]; *p* = 0.027, z = −2.21, Wilcoxon rank-sum test; and for the D1 cohort; discrimination: 0.68 [0.37–0.86]; generalization: 0.76 [0.49–0.89]; *p* = 0.01, z = −2.58, Wilcoxon rank-sum test). Stronger context remapping in reward fields during the discrimination paradigm may not be surprising given the known relationship between reward structure in behavioral tasks and place coding.^52^ Specifically, here reward outcome is tied to context in the discrimination paradigm but not in the generalization paradigm. To test whether stronger remapping in discrimination was arising because reward fields were more active in the context that was rewarded, we considered correct trials only and divided reward fields into three categories based on their activity profiles: match, neutral, and mismatch. Using fields located at the reward site rewarded in context A as an example, the categories were defined as follows: “match” indicated fields near reward A that increased rate during foraging in context A, “neutral” indicated fields near reward A that had equal rates during foraging in context A and B, and “mismatch” indicated fields near reward A that increased rate during foraging in context B. Interestingly, ~20% of reward fields experienced higher rates in the context opposite to the one that they were rewarded in (Figure 5E, mismatch), indicating that the context coding of reward locations is not always tied to positive reward expectation. In contrast to our results when including all place fields, the magnitude of reward field remapping was not impacted by experience (Figures 5F and 5G), and notably, the reward fields in the generalization paradigm exhibited the same stability between contexts, regardless of which paradigm was experienced first (Figure 5G).

### Hippocampal directional codes are not influenced by paradigm

**Figure 6.**
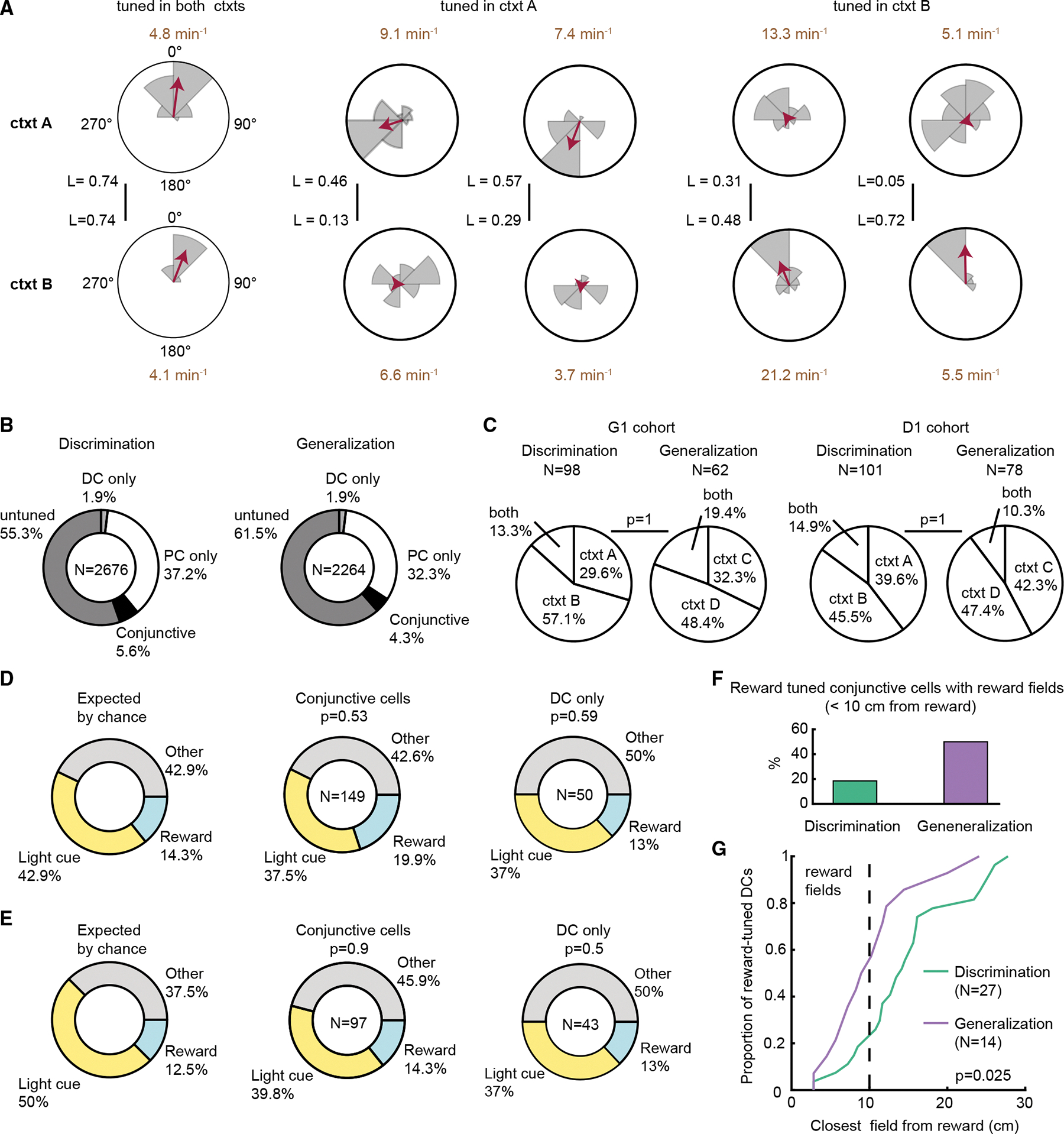
Hippocampal vector code is not influenced by context relevance (A) Example of five hippocampal directional modulated cells (DCs). Tuning curves are shown for context A (top row) and context B (bottom row). Angular direction was divided into eight bins, such that each bin represents one wall of the arena. Tuning curve (gray) and corresponding Rayleigh vector (red) are shown for each cell in each context. Vector length (L) is displayed in black, and peak event rate is displayed in brown text. (B) Composition of tuned cells. A small portion of cells were exclusively heading direction tuned (DC only), while most of the DCs were also place cells (conjunctive cells). (C) Pie charts show the proportion of DCs tuned to each context or both for G1 and D1 cohorts (*p* = 1, *p* = 1, chi-squared test). (D and E) Local landmarks were classified as “light cue,” “reward,” or “other.” The distribution of walls with each landmark was calculated to serve as the chance-level expectation for tuning (left), and the percentage of cells tuned to each landmark type was calculated for conjunctive cells (middle) and pure DCs (right). Composition of the tuning did not differ from chance level (left) in discrimination (D) or generalization (E) (chi-squared test, post hoc Bonferroni correction). (F) Portion of reward-tuned conjunctive cells in discrimination and generalization. (G) Reward coding of conjunctive cells was more strongly concentrated around the reward location in generalization (*p* = 0.025, Wilcoxon rank-sum test).

Finally, we investigated directional tuning, a vector representation that had been shown to modulate place cell activity.^53^ We calculated the running direction of the mice with respect to a reference point during locomotion (see STAR Methods for details). Tuning curves and corresponding Rayleigh vectors were calculated, and directional modulated cells (DCs) were identified (Figure 6A). A subset of cells exhibited directional tuning and most were also place cells, indicating conjunctive place-vector coding (Figure 6B). The same proportion of DCs and conjunctive cells were tuned selectively in one context for both cohorts in the discrimination and generalization paradigms (Figure 6C, G1: n.s., χ^2^ = 0.02; df = 2; D1: n.s., χ^2^ = 0.01; df = 2; chi-squared test). Cells that were tuned in both contexts did not change their angular tuning between contexts (the median angular difference was within one angular bin: 14.1° for discrimination and 12.4° for generalization). These results indicate that contexts are represented in DCs and conjunctive cells, such that the tuning is present in only one context; however, this aspect of context representation is not sensitive to paradigm type.

Given that context is defined by local landmarks, we calculated the portion of DCs that were tuned either to light cues or to reward sites. We did not find a significant modulation by local cues in either paradigm (Figures 6D and 6E, discrimination: χ^2^ = [1.3; 1] and *p* = [0.53; 0.59]; generalization: χ^2^ = [0.2; 1.4] and *p* = [0.9; 0.5]; (conjunctive cells; DC only); df = 4; chi-squared test). Next, we asked whether the reward-direction tuning was due to place field activity around the reward. We found that place activity of reward-tuned conjunctive cells is dispersed in the arena in the discrimination paradigm, while it is concentrated around the reward location in the generalization paradigm (Figures 6F and 6G; discrimination: 14.1 [10.9; 17.6] cm; median [25^th^ percentile; 75th percentile]; generalization: 9.6 [7.2; 12.2] cm; distance from reward of the closest field; z = 2.2; *p* = 0.025; Wilcoxon rank-sum test), suggesting that direction to reward is encoded at greater distances when mice have to keep track of multiple reward locations.

## DISCUSSION

Remapping between contexts was observed in a diverse set of functionally defined cell types, with context relevance impacting the magnitude of remapping in a subset. In line with previous studies, we observed robust codes for context in both place and non-place cells.^13,28,47,54,55^ Recent works propose that engram cells play an important role in context representation,^56,57^ and our finding that an SVM could discriminate between context when sampling selectively from non-place cells supports these ideas. Interestingly, only place cells were impacted by paradigm type, showing stronger separation between contexts in the discrimination paradigm compared to the generalization paradigm. This finding already hints that context relevance impacts hippocampal remapping through a selective mechanism, tweaking some but not all cells in the circuit. Such selectivity was further exemplified when we considered place cells with reward fields versus directional modulated cells. Reward modulation of place activity has been widely investigated,^52,58,59^ and we add to this body of work by showing that reward fields showed robust context relevance effects. Strong effects near rewards may implicate a role for neuromodulation as several studies have shown activation of dopaminergic input to hippocampus near rewards.^60–62^ On the other hand, DCs remapped to the same extent in both paradigms, suggesting that this cell type is impervious to context relevance. Our findings are consistent with observations that hippocampal vector coding contributes to context coding.^53,63–66^ However, in contrast to studies that show the importance of changing landmarks,^63,65,67^ we did not find evidence of vector tuning to local landmarks, suggesting that contexts in our paradigms may be perceived as a composition of distal and local cues.^7^

Prior experience impacted whether or not paradigm type modulated the magnitude of hippocampal remapping. Specifically, when animals were first trained in the high context-relevance paradigm (discrimination), hippocampal remapping continued to be stronger in subsequent tasks, even when context relevance was low (generalization). The inheritance of stronger remapping is especially interesting given that it is mapped onto distinct contexts (C/D) even though the importance of context was learned in A/B, hinting at a schema in which context has been signified as an important variable.^16,68^ Notably, the experience-dependent effects did not broadly affect all functional cell types in the hippocampus, again underscoring the idea that mechanisms governing context coding in the hippocampus are heterogeneous across cell types in the circuit. For example, context relevance remained as a modulating factor at locations near reward regardless of experience. There are several examples of experience impacting hippocampal operations^50,69^—and our results highlight that it is important to consider such experience-dependent effects when designing experiments and investigating underlying mechanisms.

There are several potential mechanisms that could support how context relevance modulates the magnitude of hippocampal remapping. As mentioned earlier, a strong candidate is neuromodulation—especially neuromodulatory circuits associated with arousal such as dopamine and noradrenaline. Manipulation experiments suggest an important role of locus coeruleus in contextual memory recognition and hippocampal remapping.^70,71^ It seems probable that the discrimination task requires more attention (i.e., higher arousal states), given that animals must attend to context-reward location pairings to successfully learn the task. Spatial representation has been shown to be modulated by the degree of attention during behavioral tasks, which potentially involves dopaminergic mechanisms in higher-order cortical regions.^72,73^ Moreover, associative learning has been shown to be dependent on dopamine in entorhinal circuits—and importantly, dopamine is necessary for schema learning such that newly learned associations are mapped onto cellular ensembles that coded previously learned associations.^74^ Perhaps a similar schema-type inheritance is at play when the animals in our study learn the generalization paradigm after the discrimination paradigm. Plasticity is likely involved, especially given the sustained effect from discrimination to generalization. Several synapses are contenders. The lateral entorhinal cortex has been shown to play a critical role in context coding, and synapses could be strengthened at subnetworks throughout the hippocampus—including the direct input from the lateral entorhinal cortex to CA1.^75,76^ Given that the discrimination task was designed to require animals to discriminate subtle difference in context (adjacent wall cues), it is also possible that pattern separation mechanisms in the dentate gyrus are at play^26,27,30–32,77,78^ and that context relevance impacts the dentate to CA3 synapse. Finally, an intriguing idea is that the mechanism involves an intersection between neuromodulation and plasticity, i.e., the presence of a neuromodulator selects particular synapses to undergo plasticity such as has been reported previously.^60,72,79^

Our results highlight that there is robust latent information in the hippocampus about context.^80^ It would be interesting to test whether the boosted remapping observed in the discrimination paradigm facilitates faster learning of new associations to the discriminated contexts. For example, would animals be faster to learn the location of a shock zone in context A (learned in discrimination) compared to in context C (learned in generalization)? Given that the strongest aspect of remapping in discrimination occurred in fields located near reward locations, it is an open question as to whether they would provide latent information for learning non-reward-based associations. Finally, we would like to stress that even in the generalization paradigm, there were strong context representations. In this case, one might have expected a single map that was bound to the invariant reward location, rather than distinct maps for each context. Instead, it seems the hippocampus is “ready” to ascribe aspects of experience to the contextual background, even when not necessary for solving the task.

### Limitations of the study

Several limitations should be acknowledged. First, we performed lesion experiments to test the role of the hippocampus in the discrimination task. A more precise approach would be the real-time manipulation of the hippocampus to dissect hippocampal functions in the task. Second, we analyzed hippocampal context coding in highly trained mice, and thus our data are limited to answer whether the extent of context coding correlates with performance and learning. Finally, we showed that one manifestation of context coding is in the rate code of place cells. However, the temporal resolution of calcium imaging is limited, and therefore the investigation of rate coding would be more precise by utilizing electrophysiological recordings.

## RESOURCE AVAILABILITY

### Lead contact

Requests for further information and resources should be directed to and will be fulfilled by the lead contact, Laura A. Ewell (lewell@hs.uci.edu).

### Materials availability

This study did not generate new unique reagents.

### Data and code availability

- Code and processed data supporting the current study are publicly available.
- Processed data are deposited on Mendeley Data: https://doi.org/10.17632/52zpg4j794.1.
- Codes supporting the current study are publicly available: https://doi.org/10.5281/zenodo.17488388.
- Any additional data are available upon request to the lead contact.

## STAR★METHODS

### EXPERIMENTAL MODEL AND STUDY PARTICIPANT DETAILS

All experimental procedures were performed as approved by the Institutional Animal Care and Use Committee at the University of California, Irvine. All the experiments were performed using 3–5 months old male and female C57BL/6 mice (Charles River). Mice were single housed and kept in an inverted 12 h light/dark cycle, with a temperature of (22 ± 2)°C and relative humidity of (55 ± 10)%. Food and water were available *ad libitum*, except during the behavioral experiments when mice received a 2% citric acid (CA) water replacement. Weight was monitored and maintained above 85% of the original weight during CA water replacement. Pain was minimized and all efforts were made to reduce the number of animals used.

### METHOD DETAILS

#### Behavioral arena

We built an octagonal-shaped arena with a radius of 20 cm, using eight custom-designed clear acrylic plexiglass walls (Ponoko, Figure S1A). Each wall was equipped with a Mouse Behavior Port (Sanworks, #1009), that contained an IR emitter and sensor, a solenoid valve, and an LED light. Additionally, a 15 cm long LED strip cue was mounted diagonally above the port on each wall. Light cues and reward ports were then wired to an Arduino Mega 2560 to allow direct control of them by custom-written Arduino scripts. Ports were calibrated to deliver 10 μL of liquid reward (5% sucrose). The arena was surrounded by distal visual cues, two speakers (LogiTech Z150) and a box insulated with acoustic foam. A web camera (TECKNET 1080p Webcam) was mounted on the ceiling of the box, centered above the arena.

#### Behavioral task

In the discimination paradigm, mice were trained to discriminate between two similar contexts that were defined by adjacent light cues, to retrieve reward at two different locations. In the generalization paradigm, mice were trained to associate two similar contexts with one reward location. We implemented a custom-written Bonsai Rx^81^ workflow to control our behavioral task and to acquire, synchronize and process distinct data streams in real-time (Figures S1B and S1C). Each trial started with illumination of light cues (which defined contexts) and the definition of a randomized hidden trigger zone (7 × 7 cm). The first phase of the trial is considered ‘foraging’ and is always associated with a specific context. The reward retrieval phase was started when the mouse center of mass (tracked in real time with Bonsai) entered the trigger zone (at least 10 s after the start of the trial). The reward retrieval phase was accompanied by a 10 kHz sound tone (Figure 1C). During the foraging phase, the mouse was allowed to visit any port (IR beam breaks were signaled to Bonsai via the Arduino). Trials were classified into three types based on actions during the reward retrieval phase: (1) Correct trials; the mouse visited the correct port during the reward retrieval phase and liquid reward was delivered, (2) Incorrect trials; the mouse visited any other port during the reward retrieval phase and flashing lights indicated trial end, and (3) Timed out trials; the mouse did not visit any of the ports during the reward retrieval phase within 1 min and flashing lights indicated trial end. In all cases, upon trial termination, the sound tone switched off and the next trial started after a 3 s inter-trial interval. Trials were pseudo-randomized such that context identities were balanced and intermingled.

#### Training protocol

Mice were single-housed and regular water was replaced by 2% citric-acid water^82^ two days before any behavioral experiment (Figure S1D). Each behavioral session was 1 h long, allowing the mice to run 70–100 trials. Mice were first habituated to the arena and to the liquid reward for two days. Next, mice were pre-trained either on the discrimination paradigm (6 males and 9 females), or on the generalization paradigm (3 males and 1 female). During pre-training, the LED of the rewarded port was lit during reward retrieval phase to guide the mouse to the correct port (cued trials). On the first two days of pre-training, mice were allowed to visit any port during reward retrieval. From the third day, visiting non-rewarded ports during the reward retrieval phase was considered as an incorrect trial. Mice were transferred into the training phase if they had at least 70% of correct trials. During the training phase, 8 cued trials were followed by 24 non-cued trials, in which mice were required to find the reward without the guide of the LED of the rewarded port. We considered that mice had learned the task when they reached a 70% performance criteria on the non-cued trials. One group of mice that learned to discriminate contexts first (D1 cohort) were then trained on the generalization paradigm without additional pre-training, until they reached the 70% criteria. Another group of mice learned to generalize contexts first (G1 cohort) and then were pre-trained on the discrimination paradigm for two days and then trained until they reached the 70% criteria. Note that we varied the reward locations among mice in both learning paradigms.

#### Surgical procedure

For all surgeries, 10–14 weeks old mice were anesthetized with 1–2% isoflurane and placed into a stereotaxic frame (David Kopf Instruments). Buprenorphine (Patterson Veterinary, 0.1 mg/kg) was injected to minimize discomfort and mice were kept on a heat pad to maintain body temperature. After surgery, Carprofen (MWI Veterinary supply, 5 mg/kg) was administered subcutaneously for the next 3 days and weight was monitored for 7 days.

For the hippocampal lesion experiments, either colchicine (Sigma-Aldrich, C9754, 2.5mg/ml) or 0.9% NaCl was injected bilaterally into dorsal hippocampus (AP: −2.0 mm, ML: ±1.25 mm from bregma; DV: −1.5 mm from dura; 125 nL/injection site).

For the calcium imaging experiments, pGP-AAV-*syn*-jGCaMP7f-WPRE (Addgene, 100 μL at a titer of ≥ 1×10^13^ vg/mL) was diluted in 0.9% NaCl (1:4 ratio) and injected unilaterally into dorsal CA1 at two sites (AP: −1.9 mm, ML: −1.5 mm and −1.6 mm from bregma, DV: −1.1 mm from dura; 300nL/injection site). Two weeks later, a second surgery was performed and a 2 mm diameter craniotomy was made above the hippocampus on the right hemisphere (centered at AP: −1.9 mm, ML: −1.5 mm). Cortical tissue was removed using a blunt 27G needle connected to a vacuum pump until the alveus fibers above CA1 were visible through a Leica M80 microscope, and a 1 mm diameter GRIN lens (Go!Foton, CLHS100GFT003) was implanted and secured with dental cement. Five days later the field of view was visualized using a UCLA v4 miniscope (https://github.com/Aharoni-Lab/Miniscope-v4) that was attached to a baseplate. Once a clear field of view was found, the baseplate was secured to the implant with dental cement. The dental cement was painted with black nail polish to prevent light contamination during experiments.

#### Hippocampal lesion experiment

We trained 12 mice on the discrimination paradigm. Upon reaching the learning criteria, mice were given regular water. The next day mice were bilaterally injected either with colchicine (*n* = 6) or with 0.9% NaCl (*n* = 6) into dorsal hippocampus. Both the lesion and control groups were balanced for sex. 12 days post-surgery, mice received CA water again and two days later were retested on the discrimination paradigm for four consecutive days. Similar to the training phase, non-cued trial blocks were interleaved with cued trial blocks on each day. Mice were then intracardially perfused with 4% paraformaldehyde (PFA) solution. Brains were extracted and stored in 4% PFA. 48 h before sectioning, brains were transferred into 30% sucrose solution (diluted in 1X phosphate-buffered saline) and 50 μm coronal sections were cut throughout the entire hippocampus using a microtome. Slices were then Nissl-stained for visualization of cell bodies and TIMM-stained for visualization of mossy fibers. Dorsal hippocampus was considered from the region where the two blades of DG became visible (AP −1.2 mm from bregma in control mice) to the region where ventral CA1 appeared (AP: −2.5 mm from bregma in control mice). An investigator manually measured the area of dorsal DG, CA3 and CA1 of every third slice on both hemispheres, for 5 control mice (brain of one control mouse was damaged and not used for this analysis). Areas were then summed up and interpolated between slices. Average across mice and hemispheres was considered as the baseline volume for each region. Next, the same measurement was repeated for each lesioned dorsal hippocampi by two investigators. Results from the two investigators were then averaged. Finally, the volume of each region was compared to the baseline value to calculate a relative dorsal hippocampal volume.

#### Calcium imaging sessions

A UCLA v4 miniscope was secured to the baseplate fixed to the skull of the mouse and connected to a commutator (Open Ephys, OEPS-7759) that was centered above the arena. Data was acquired with a Miniscope DAQ board (https://github.com/daharoni/Miniscope_DAQ_PCB). Focal plane and light excitation intensity was set before each imaging session. Miniscope and commutator were controlled in a custom-written Bonsai workflow. Only sessions with 70% or higher behavioral performance were included in the analysis and the recorded cells were not tracked between the discrimination and generalization paradigms.

### QUANTIFICATION AND STATISTICAL ANALYSIS

#### Position and speed analysis

Mouse behavior was recorded with a web camera (TECKNET 1080p Webcam) with a frame rate of 30 fps. For online position estimation, the center of mass of the mouse was calculated in Bonsai RX. For later analysis, the position of the head and body was post-hoc tracked in DeepLabCut.^83^ Speed was calculated using the position of the body that was smoothed with a 167 ms wide moving average filter. Any further spatial analysis was performed on the position of the head.

#### Preprocessing calcium imaging data

Minian^84^ was used to extract spatial footprints, their corresponding calcium traces and deconvolved signals. Next, we determined whether the baseline of the calcium trace was stable during the recording session. We segmented the calcium traces into one minute long sections and measured the valley of each segment. If the valley exceeded 0.1 ΔF/F_0_ for at least 5 min, the calcium trace was labeled as an unstable signal. The labels were then manually verified in a custom-written graphical user interface (GUI) by inspecting individual calcium traces and deconvolved signals. Cells with unstable calcium dynamics were excluded from further analysis (see Figure S2). To determine calcium event timings of the accepted units, peaks of the deconvolved signals were detected.

#### Population decoding

Analysis was restricted to the foraging phase of the trials, including running and resting epochs. Start of the foraging phase was defined by the first active locomotion in the trial. The calcium event time series for each cell (sampled at 20 frames per second) was smoothed using a sum of calcium events over non-overlapping windows of 60 frames, resulting in a new time series with the same sample rate. The time series was restricted to frames where foraging was performed. Finally, to reduce computational complexity, only every 20th frame (i.e., 1 sample/second) was fed into the decoder. The binned calcium activity was used to train a support vector machine (SVM) with a radial basis function kernel. The data was standardized to a standard normal distribution (*Z* score) as a pre-processing step in the MATLAB (2024b) function fitcsvm(). 80% of the frames were used for training, and 20% were used as a testing set to assess overfitting. To tune the kernel scale hyperparameter, 20-fold cross-validation was used. The SVM was trained separately for each animal and each task to predict the binary “context” during each foraging frame. Furthermore, for each animal and task, SVMs were trained using three cell subsets: (1) all cells, (2) place cells only, and (3) non-place cells only. In all cases, the mean cross-validated classification accuracy scores were reported as a measure of context decoding performance. To assess the influence of cell-type composition and task demand on decodability of context, in MATLAB, a repeated measures model was specified by calling fitrm() with a between subject model of a constant mean, and within subject factors of cell-set type (PCs versus non-PCs) and task (discrimination versus generalization) as separate categorical variables. Then, ranova() with within model CellSet +Task was called to estimate model fit. The resulting model was used to compute conditional marginal means with the MATLAB multcompare() function, comparing decoder accuracy by task between cell types. P-values were corrected using the Tukey’s multiple comparisons.

#### Place cell identification

For each neuron, calcium event trains and mouse position data during the foraging phase of each trial were isolated into ‘foraging segments’. Only periods during running epochs (speed exceeded 2 cm/s) were included. Next, the foraging segments associated with each context were concatenated such that activity maps for individual contexts could be calculated. To create context place maps (i.e., each neuron had two maps), position was divided into 2 × 2cm and calcium event rates were calculated in each bin. The place maps were smoothed by a Gaussian-filter (σ = 3 cm). To test for spatial stability of place firing, we split maps into the first and second in time or into even and odd trials. Spatial rate maps were constructed for each condition and Pearson’s correlation was calculated for first half vs. second half maps and even vs. odd maps. For each context-map, the average of the two correlation values was taken as a measure of spatial stability. Calcium event times were then circularly shuffled (at least ±1 min away from the original timepoints) and the correlation coefficients were recalculated 500 times to create a null distribution. For statistical analysis of spatial stability, the Fisher’s transformation of the Pearson’s correlation was used. A cell was considered as a place cell if its spatial stability exceeded the 95th percentile of the null distribution and the value was higher than 0.4, at least in one context. Place cells firing less than 10 times during the entire session were excluded.

#### Place field identification

Calcium events of the two contexts were pooled and the ‘full’ rate map of each place cell was recalculated. To determine the number of place fields of each rate map, peaks with at least 1 event/min prominence were detected in MATLAB using the *islocalmax2* function. In order to define the boundaries of each place field, a k-component Gaussian mixture model was fitted on the rate map, where k is the number of place fields. Pixels of the fields lower than 20% of the peaks were excluded. Next, the detected place fields and the rate maps were manually inspected in a custom-written GUI to readjust field boundaries or isolate fields that were not captured by the algorithm, when needed. Finally, the spatial bins of each field were used to calculate field parameters, such as mean rate, field size and field center. Place fields with less than 1 event/min peak or with less than two events were excluded from further analysis.

#### Rate analysis

For each field, rate remapping was measured by calculating the rate overlap score of the mean field rate between the two contexts, defined as

$$\mathrm{Rate}\;\mathrm{Overlap}\;\mathrm{Score}=1-\frac{|{\mathrm{rate}}_{1}\;-\;{\mathrm{rate}}_{2}|}{{\mathrm{rate}}_{1}+{\mathrm{rate}}_{2}}$$

Fields with a field center 10 cm or less from reward locations were considered as ‘reward fields’. Note that field activity comprises time during the foraging phase of each trial and does not include activity during reward consumption. For the mismatch analysis, calcium events during correct non-cued trials were used only and rate maps were recalculated for each context. Firing preference of the reward fields was defined as

$$\mathrm{Firing}\;\mathrm{Preference}=\frac{{\mathrm{rate}}_{1k}\;-\;{\mathrm{rate}}_{2k}}{{\mathrm{rate}}_{1k}+{\mathrm{rate}}_{2k}}\;*\;{\left(-1\right)}^{k+1}$$

where k∈{1,2} is the reward location where the field is located. Reward fields were considered as “match” fields if its firing preference exceeded 0.33 and as “mismatch” fields if the firing preference was lower than −0.33. The remaining reward fields were classified as “neutral”. Fields further than 10 cm from reward were further divided into 5 cm increments of the field center-reward location distance. In the case of the discrimination paradigm where two rewards were present, the closer distance was considered for each field.

#### Directional analysis

Similarly to the spatial analysis, analysis was restricted to the foraging phase of the trials during running epochs. Relative running direction to a chosen reference point was calculated based on.^65^ Briefly, position of the head was smoothed with a 167 ms wide moving average filter and the mouse’s running direction was calculated as

$$\varphi_{j}={tan}^{-1}\frac{y_{i+1}\;-\;y_{i}}{x_{i+1}\;-\;x_{i}}$$

where ($x_{i};y_{i}$) are the coordinates of the mouse’s head at time i. Next, the angle between the reference point and the mouse’s head was measured as

$$a_{i}={tan}^{-1}\frac{RY\;-\;y_{i}}{RX\;-\;x_{i}}$$

where (RX;RY) are the coordinates of the reference point. Finally, the relative running direction was defined as the difference of the two angles

$$H_{i}=\mathrm{modulo}\left(\left(\varphi_{i}-a_{i}\right)+\pi ,2\pi \right)-\pi$$

Running direction was divided into 45° wide bins, such that each bin corresponds to one wall on the arena. Tuning curves were created by calculating the calcium event rate in each angular bin for each context and the Rayleigh’s vector was calculated. Strength and direction of the tuning were considered as the length and angle of the Rayleigh’s vector. Next, the session was split into first and second half, and even and odd trials and the tuning curves were recalculated for each context. Pearson’s correlation of the tuning curves was calculated between first and second half trials, and even and odd trials, then averaged. Tuning curves were considered as stable if the correlation coefficient exceeded 0.4 at least in one context. Next, calcium events were circularly shuffled 500 times and the Rayleigh vectors for each context were recalculated to create a null distribution. A cell was considered as significantly directional tuned if the Rayleigh’s vector length exceeded the 95th percentile of the null distribution at least in one context. We further performed a second shuffling procedure, in which the running direction was shuffled.^64^ To ensure that directional analysis were not biased by non-uniform positions within the environment (i.e., more time against walls that prohibit certain heading directions), we implemented a reconstruction analysis based on Sarel et al. In this measure, directional tuning curves were reconstructed with the assumption that the cell was a pure place cell using the following equation:

$$r^{\prime }(H)=\frac{\Sigma_{x,y}(p(x,\;y,\;H)\;*\;r(x,\;y))}{\Sigma_{x,y}p(x,\;y,\;H)}$$

Where p(x,y,H) is the probability that the mouse was in the x,y spatial bin with a direction of H, and r(x,y) is the observed spatial rate map. Next, we reconstructed the spatial rate map assuming pure directional tuning of the cell as the following:

$$r^{\prime }(x,\;y)=\frac{\Sigma_{H}\;(p(x,y,\;H)\;*\;r(H))}{\Sigma_{H}\;p(x,\;y,\;H)}$$

where r(H) is the observed directional rate map. For both directional and spatial reconstructed rate maps, we calculated the error as

$$\mathrm{Error}=\frac{\left\langle {\left(\mathrm{observed}\;-\;\mathrm{reconstructed}\right)}^{2}\right\rangle }{max(\mathrm{observed})\;-\;min(\mathrm{observed})}$$

where both the observed and reconstructed rate maps were normalized to their peak rates. Finally, we defined the direction/place index as

$$\mathrm{Direction}\;/\;\mathrm{place}\;\mathrm{index}=\frac{\mathrm{error}\;\mathrm{assuming}\;\mathrm{pure}\;\mathrm{place}\;\mathrm{cell}\;\mathrm{tuning}}{\mathrm{error}\;\mathrm{assuming}\;\mathrm{pure}\;\mathrm{goal}\;\mathrm{tuning}}$$

A cell was considered as a directional modulated cell (DC) if its tuning curve was stable, the tuning strength was significant after both of the shuffling procedures and the direction/place index exceeded 1 in at least one context. Cells that were identified both as place cells and directional modulated cells, are referred as “conjunctive cells”. Cells with less than 10 calcium events were excluded.

Tuning difference was calculated for DCs that were tuned in both contexts by taking the difference of the tuning directions in the two contexts. To analyze tuning to local cues, tuning was categorized into “reward”, “light cue” or “other”. For the discrimination, one of the light cues and a reward location share the same bin, therefore this bin was excluded (varies among mice). To measure field distance of reward tuned conjunctive cells, each field center of the cell was measured and the closest one to the reward was used.

### Statistical analysis

Statistical analysis was performed in MATLAB except for Figure 1G, which was done in GraphPad Prism. Prior to statistical comparison, normality was tested with the Anderson-Darling test and either parametric or non-parametric statistical test was used accordingly. Statistical tests included unpaired *t* test, one-way ANOVA and repeated measure two-way ANOVA (Figures 1 and S1), paired *t* test and repeated measures (Figures 2 and S3), Wilcoxon rank sum tests (Figures 3, 4, 5, and 6) and chi-squared test (Figure 6). Post-hoc Bonferroni or Tukey’s multiple comparisons was applied when required.

## Supplementary Material

1

SUPPLEMENTAL INFORMATION

Supplemental information can be found online at https://doi.org/10.1016/j.celrep.2025.116682.

## Figures and Tables

**KEY RESOURCES TABLE:** 

REAGENT or RESOURCE	SOURCE	IDENTIFIER

Bacterial and virus strains		

pGP-AAV-*syn*-jGCaMP7f-WPRE	Addgene	104488-AAV1

Chemicals, peptides, and recombinant proteins		

Colchicine	Sigma-Aldrich	C9754
Cresyl-violet for Nissl staining	Millipore Sigma	1.05235.0025
Citric-acid	Sigma-Aldrich	C0759-1KG

Deposited data		

Processed data	this paper	https://doi.org/10.17632/52zpg4j794.1

Experimental models: Organisms/strains		

C57BL/6	Charles River	027

Software and algorithms		

MATLAB 2024	Mathworks	https://www.mathworks.com/products/matlab.html
Prism	GraphPad Software	https://www.graphpad.com/
Bonsai RX	Lopes et al.^81^	https://bonsai-rx.org/
Arduino	Arduino	https://store-usa.arduino.cc/
Custom code	this paper	https://doi.org/10.5281/zenodo.17488388

Other		

UCLA miniscope v4	OpenEphys	OEPS-7407
Miniscope DAQ v 3.3	OpenEphys	OEPS-7431
Miniscope v4 Coaxial Cable	OpenEphys	OEPS-5503
Mouse Behavior Port	Sanworks	1009
Miniscope v4 Baseplate	OpenEphys	OEPS-7415
Commutator	OpenEphys	OEPS-7759

## Highlights

- CA1 neuronal population codes for contexts in a discrimination/generalization task
- Remapping is stronger when context is relevant to solve the memory task
- Place cells and specifically reward-tuned cells are modulated by context relevance
- Cells with directional tuning encode context but not context relevance
