# Supplementary material for: The relevance of context in memory tasks influences the magnitude of hippocampal remapping
Source: Cell Rep. Author manuscript; Available in PMC 2026 Jan 27. (PMC12840010; doi:10.1016/j.celrep.2025.116682)
Supplement: 1 [file NIHMS2132772-supplement-1.pdf]

Cell Reports, Volume *44*

Supplemental information

The relevance of context in memory tasks influences the magnitude of hippocampal remapping

Gergely Tarcsay, Nicola Masala, Justin D. Yi, Mari K. Igarashi, Usean J. Redic, and Laura A. Ewell

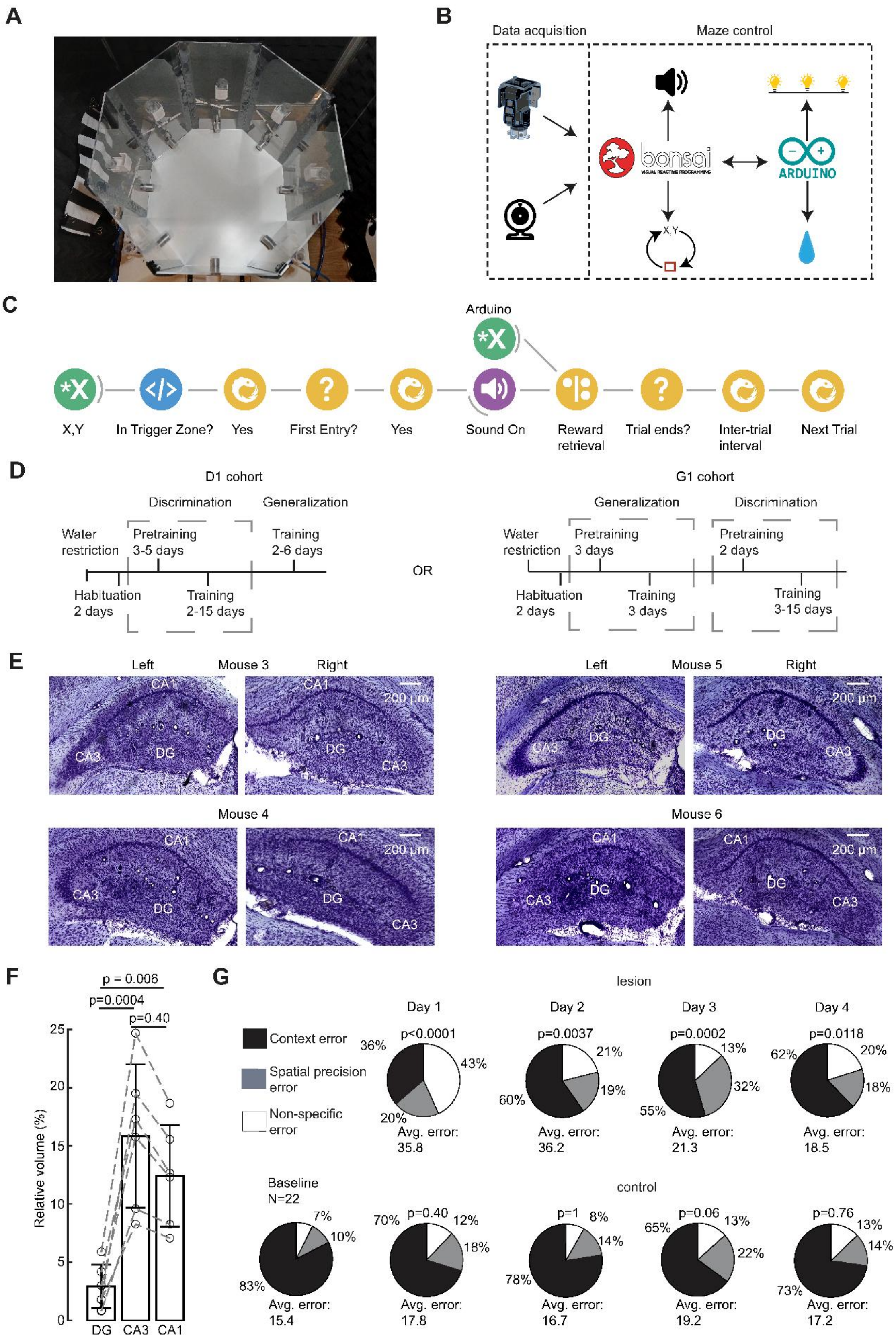

A
B
Data acquisition
Maze control
bonsai
ARDUINO
X,Y
C
Arduino
X,Y
In Trigger Zone?
Yes
First Entry?
Yes
Sound On
Reward retrieval
Trial ends?
Inter-trial interval
Next Trial
D
D1 cohort
Discrimination
Generalization
Water restriction
Pretraining 3-5 days
Training 2-6 days
Habituation 2 days
Training 2-15 days
OR
G1 cohort
Generalization
Discrimination
Water restriction
Pretraining 3 days
Pretraining 2 days
Habituation 2 days
Training 3 days
Training 3-15 days
E
Left
Mouse 3
Right
Mouse 4
Left
Mouse 5
Right
Mouse 6
CA1
CA3
DG
200 µm
F
p = 0.006
p=0.0004
p=0.40
Relative volume (%)
DG
CA3
CA1
G
lesion
Day 1
Day 2
Day 3
Day 4
Context error
Spatial precision error
Non-specific error
p<0.0001
36%
43%
20%
Avg. error: 35.8
p=0.0037
21%
60%
19%
Avg. error: 36.2
p=0.0002
13%
55%
32%
Avg. error: 21.3
p=0.0118
62%
20%
18%
Avg. error: 18.5
control
Baseline N=22
7%
10%
83%
Avg. error: 15.4
p=0.40
70%
12%
18%
Avg. error: 17.8
p=1
8%
14%
78%
Avg. error: 16.7
p=0.06
65%
13%
22%
Avg. error: 19.2
p=0.76
13%
14%
73%
Avg. error: 17.2

Figure S1. Schematics of the hippocampal-dependent discrimination task.
(A) Picture of the octagonal-shaped arena. Each wall was equipped with a reward port and with a strip of LEDs.
(B) Schematics of data acquisition and maze control.
(C) Example of the Bonsai workflow. Mice were tracked in real-time (X, Y). When the mouse entered the random hidden trigger zone, a sound tone turned on to indicate the start of the reward retrieval phase. An Arduino event flag was sent when the trial ended and the next trial started after a short inter-trial interval.
(D) Schematics of the training protocols. D1 mice were trained on the discrimination paradigm first (left). G1 mice were trained on the generalization paradigm first (right).
(E) More examples of hippocampal lesions.
(F) Relative volume of lesioned dorsal DG, CA3 and CA1 compared to baseline volumes (averaged across control mice and hemispheres)(one-way ANOVA). Data are presented as mean ± s.d.
(G) Classification of incorrect trials into ‘context error’ (visits port that is rewarded in the other context); ‘spatial precision error’ (adjacent port to reward port); or ‘non-specific error’ (any other port)(repeated measure two-way ANOVA). Baseline was calculated using 22 trained mice. The average number of errors are displayed below pie charts.
Related to Figure 1.

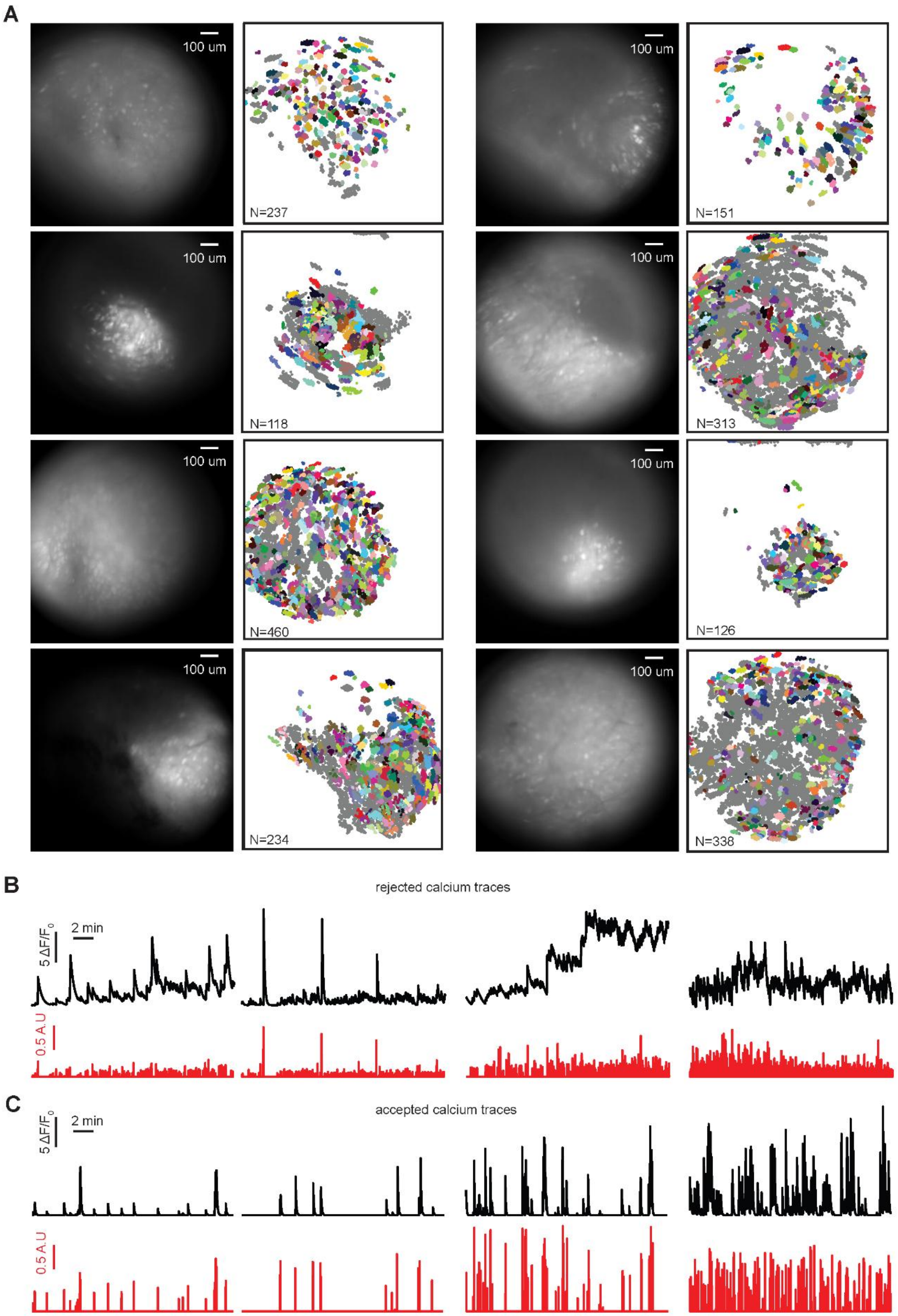

Figure S2. Example field of views and recorded calcium traces.

(A) Hippocampal cranial windows and extracted spatial footprints for the 8 additional animals (the 9th is shown in the main text) included in this study. Rejected units are shown in gray.
(B) Calcium traces (top, black) and deconvolved signals (bottom, red) for four rejected units. Note that unstable calcium dynamics introduced noise in the deconvolved signal.
(C) Same as in (B), but for four accepted units.
Related to Figure 2 and STAR methods.

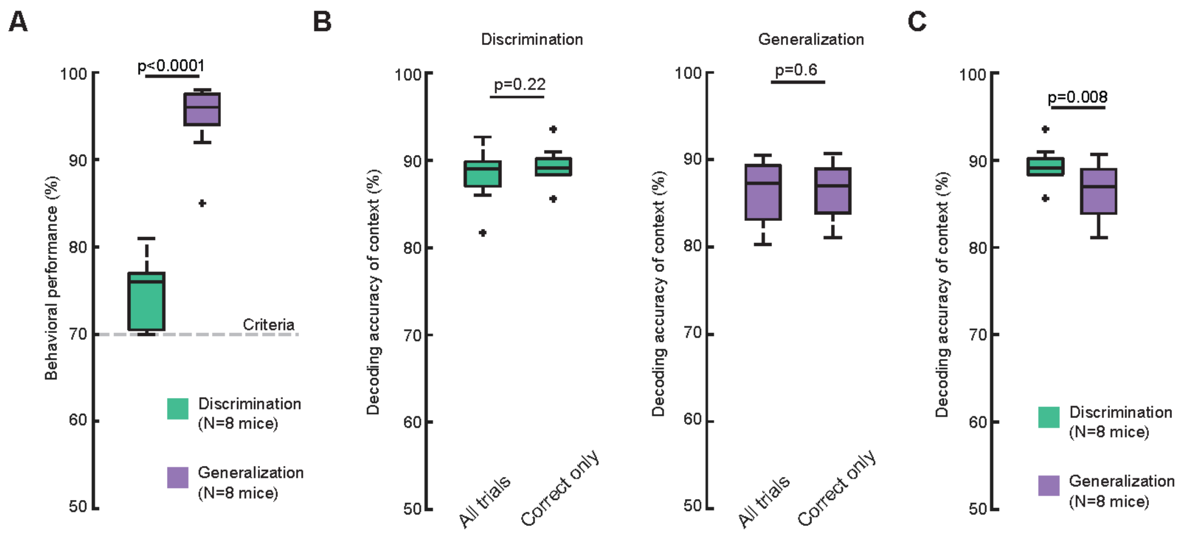

Figure S3. Decoding accuracy did not depend on behavioral differences.
(A) Behavioral performance in the discrimination (green) and generalization (purple) paradigms of the mice used for the SVM decoding analysis (paired t-test). Data are presented in box plots showing median and interquartile ranges.
(B) Comparison of decoding accuracy of contexts when SVM was trained on all trials and when training was restricted to correct trials only both for discrimination (left) and generalization (right) (paired t-test with post hoc Bonferroni correction).
(C) Decoding accuracy of context between discrimination and generalization when SVM was trained on correct trials exclusively (paired t-test).
Related to Figure 2.

Supplementary Tables

Source of variation	Sum of squares	Degree of freedom	Mean square	F-stat	P-value
Brain region	538.76	2	269.4	13.37	0.00046
Error	302.16	15	20.1	-	-
Total	840.92	17	-	-	-

Table S1. One-way ANOVA table for relative volume of lesioned hippocampus. Related to Figures 1 and S1.

Comparison	Mean difference	95% C.I.	P-value

DG vs CA3	-12.9	-19.7 to -6.2	0.0004
DG vs CA1	-9.5	-16.2 to -2.8	0.006
CA3 vs CA1	3.4	-3.3 to 10.2	0.40

Table S2. Post-hoc Tukey's multiple comparisons test between lesioned brain regions. Related to Figures 1 and S1.

Source of variation	% of total variation	Sum of squares	Degree of freedom	Mean square	F-stat	P-value
Time x Group	18.31	2336	4	583.9	F(4,40)= 7.67	0.0001
Time	37.05	4726	4	1181	F(4,40)= 15.52	< 0.0001
Group	1.945	248.1	1	248.1	F(1,10)= 1.033	0.3335
Subject	18.83	2402	10	240.2	F(10,40)= 3.156	0.0046
Residual	-	3045	40	76.13	-	-

Table S3. Repeated measure two-way ANOVA table for the hippocampal lesion experiment. Related to Figures 1 and S1.

Comparison	Mean difference	95% C.I.	P-value
lesion vs control criteria passed	-3.5	-15.60 to 8.604	0.564
lesion vs control Test Day 1	23.5	11.40 to 35.60	0.0003
lesion vs control Test Day 2	14.17	2.063 to 26.27	0.0227
lesion vs control Test Day 3	-6.5	-18.60 to 5.604	0.2859
lesion vs control Test Day 4	-7.333	-19.44 to 4.771	0.2294
Criteria passed vs Test Day 1 lesion	38.67	24.28 to 53.05	<0.0001
Criteria passed vs	26.67	12.28 to 41.05	<0.0001

Test Day 2 lesion			
Criteria passed vs Test Day 3 lesion	7.67	-6.72 to 22.05	0.55
Criteria passed vs Test Day 4 lesion	4.17	-10.22 to 18.55	0.92
Test Day 1 vs Test Day 2 lesion	-12	-26.39 to 2.39	0.14
Test Day 1 vs Test Day 3 lesion	-31	-45.39 to -16.61	<0.0001
Test Day 1 vs Test Day 4 lesion	-34.5	-48.89 to -20.11	<0.0001
Test Day 2 vs Test Day 3 lesion	-19	-33.39 to -4.6	0.0045
Test Day 2 vs Test Day 4 lesion	-22.5	-36.89 to -8.11	0.0006
Test Day 3 vs Test Day 4 lesion	-3.5	-17.89 to 10.89	0.96

Table S4. Post-hoc Tukey's multiple comparisons test for performance between lesion and control group. Related to Figures 1 and S1.

Mouse ID	All cells		Place cells only		Non-place cells only	
	D	G	D	G	D	G
M210	460	-	242	-	218	-
M119	237	159	157	104	80	55
M120	151	168	100	96	51	72
M292	118	112	49	41	69	71
M319	313	476	86	106	227	370
M231	126	145	38	38	88	107
M314	554	537	241	274	313	263
M316	234	141	74	41	160	100
M318	338	398	155	129	183	269

Table S5. Number of cells for each mouse in the discrimination (D) and generalization (G) used in the decoding analysis. Note, mouse 210 was not run in the generalization paradigm

and was not included in the SVM analysis or statistics presented in the main results. Related to Figure 2.

Mouse ID	All cells all trials		Place cells only		Non-place cells only		All cells; Correct trials	
	D	G	D	G	D	G	D	G
M210	94.95	-	92.98	-	90.05	-	95.38	-
M119	89.46	85.76	88.20	83.63	64.95	63.00	88.37	85.83
M120	88.07	88.89	86.32	85.10	72.81	75.06	89.22	88.46
M292	85.99	80.57	85.20	74.05	73.68	70.82	88.32	81.09
M319	89.68	90.49	86.94	86.19	86.00	87.95	90.95	90.68
M231	81.70	80.26	73.86	71.22	74.09	73.20	85.62	81.95
M314	92.68	89.79	91.01	88.99	87.29	81.15	93.57	89.47
M316	88.53	86.94	81.45	77.45	84.95	80.80	88.96	86.09
M318	90.03	87.61	86.73	84.04	80.92	81.99	89.39	87.92

Table S6. Decoding accuracy for each mouse in the discrimination (D) and generalization (G). Four cases were investigated: 1) decoding with all cells; 2) decoding with only place cells; 3) decoding without place cells (Figure 2D and 2G); and 4) decoding with all cells but restricted to correct trials (Figure S3). Note, mouse 210 was not run in the generalization paradigm and was not included in the SVM analysis or statistics presented in the main results. Related to Figures 2 and S3.

	Sum of squares	df	Mean square	F	p-Value
Intercept	20.625	1	20.625	1.818e+03	1.02e-09
Error	0.079	7	0.011	1	0.5
Intercept: CellSets	0.026	1	0.026	3.89	0.089
Error (CellSets)	0.047	7	0.007	1	0.5
Intercept:Tasks	0.005	1	0.005	7.21	0.031
Error (Tasks)	6.84e-04	7	6.84e-04	1	0.5

Table S7. Repeated measures model for decoding accuracy. Related to Figure 2.

Comparison	Mean difference	Standard error	95% C.I.	P-value
Discrimination vs generalization;	3.63%	1.17%	1.34% to 5.92%	0.017

place cells				
Discrimination vs generalization; non-place cells	1.34%	1.06%	-0.74% to 3.42%	0.25
Place cells vs non-place cells; discrimination	6.88%	3.10%	0.804% to 13.0%	0.062
Place cells vs non-place cells; generalization	4.49%	2.84%	-1.08% to 10.1%	0.15

Table S8. Post-hoc Tukey's multiple comparisons for decoding accuracy. Related to Figure 2.
